# Neuralgia Treated by Black Snake Root

**Published:** 1852-07

**Authors:** 


### SELECTIONS.

From the Western Lancet.

### *Neuralgia treated by Black Snake Root.*

I SEND you from my *register*, the history of half a dozen cases of Neuralgia, treated by the tincture of black snake root. I commenced using this article about two years since, in the case of Mrs. Mc—— after the reported articles had failed to relieve her of a periodical attack of facial neuralgia. Cathartics, quinine, arsenic, and iron, had in turn or conjointly all been used without accomplishing any permanent benefit for the patient. The pain would make its appearance in the morning, soon after sunrise, and increase in severity until mid-day, when it became so excruciating as scarce to be endured by the patient. This state of affairs would continue for an hour or two, and the pain would then gradually diminish in intensity until evening, when she became comparatively comfortable.

At this time, December 8th, 1850, the tongue was covered with a thick white coat, bowels open, pulse accelerated, and during the exacerbations, they became full with some force. The parts affected were tumid, above natural temperature, and exceedingly sensitive; intolerance of light and sound, and the eye of the affected side suffused and injected.

I began by directing the patient to take a dram of the tincture of black snake root every six hours, and persisted in its use for twenty-four hours, without much abatement of the painful symptoms. The same amount of medicine was still continued, but at intervals of four hours, with marked improvement in the condition of the patient by the next daily visit. A mercurial cathartic was now administered, to correct the depraved secretions, and unload the bowels. The other medicine was continued *ut supra.*

On the afternoon of the following day, December 11th, I found that the cathartic had acted briskly, and the patient had missed the paroxysm, the first time for thirteen days. But there was a sense of confusion in the head, and a dull, heavy pain in the occipital region, with a dragging sensation in the external ear. The following day, the patient took four drachm doses of the medicine, without any return of the paroxysm. Medication was now suspended, and the cerebral symptoms soon disappeared.

CASE II, was intercostal neuralgia, of three years standing in the person of Mrs. W.; it was periodical in its nature. Almost every article of the Materia Medica which promised any prospect of relief, had from time to time been brought to bear upon the case, with but temporary relief. After correcting the secretions, the tincture of macrotys was administered in drachm doses, every five hours for three days, before the patient was perfectly free from the pain, it having commenced to decrease after the first thirty hours. She took the same amount of medicine three times per day for a week; since when, the patient has not had a return of neuralgia.

CASE III. J. I., aged 24. Nervous temperament, has been laboring under supra-orbital neuralgia thirty-six hours. The left half of the forehead is tumid as high up as the scalp, and extending across the temple. The entire extent pervaded by the disease is of a scarlet hue, exceedingly painful and sensitive. Tongue covered with a white fur, bowels constipated, skin dry and harsh, pulse quick and corded. I directed him to take immediately, half a scruple of calomel and eight grains of Dover's powder, to be followed in three hours with an ounce of sulphate of magnesia. After the bowels moved, he was to commence and take every fourth hour, a teaspoonful of the tincture of black snake root. The next day, March 23, 1851, I learned that my patient's bowels had been freely acted upon, and that he had taken five doses of the tincture. The pain was less acute; light and currents of cold air could be endured without inducing such intense suffering as on the 22d. The same amount of medicine was directed to be continued at intervals of three hours, and ten grains of Dover's powder at bed time.

On the 25th, the patient informed me that he had rested better during the night, than any former period of the same duration, since his seizure. He expressed himself perfectly free from orbital pain; and the tumefaction had almost wholly disappeared, as well as that of the redness. The patient complained of an uneasy sensation in the upper and back part of the head, and humming in the ears; which confused and annoyed him very much. I ordered the medicine to be omitted, and directed a brisk saline cathartic. The next day I found my patient comfortable, and free from all trouble in the head.

CASE IV. Mrs. E., aged 27, sanguine temperament, has had neuralgia for five days, during which time she has been unable to take any regular sleep. Has been under the influence of hydropathic treatment from the beginning of the disease.

December 19th, 1851, I found her laboring under all the symptoms of facial neuralgia, in an aggravated form. The nerves of the right side were affected, her bowels open, skin above natural temperature and dry, pulse quick and corded. There were no diurnal mutations. I put her upon the use of the tincture of macrotys racemosa in drachm doses every three hours.

I saw her the next day, after she had taken an ounce of the medicine, and learned that the pain had gradually diminished after the fourth dose, and now she was perfectly free from all uncomfortable sensations about the head. There was no more return of the disease.

Case V. Mr. M., age 36, scrofulous diathesis. This patient has been laboring under an attack of bilious vomiting and fever for five days; but which has been controlled by the use of alterants, antiphlogistics and quinine. At present, January 15th, 1852, his tongue is clean, bowels open, skin dry and harsh, pulse quick and corded. The right side of the face and temple are of a crimson hue and considerably swollen. There is intolerance of light and sound, and the eye of the affected side is suffused and injected. A sharp, shooting pain emerges from a point just in front of the right ear, and radiates over that half of the face, brow and scalp. It is of the most acute character. Having taken a drachm and a half of quinine in arresting the fever, I deemed it unnecessary to push its use any further in this case. I therefore commenced immediately upon the administration of the tincture of black snake root. He took an ounce in twenty-four hours, in divided doses, without any beneficial results. The skin was unusually dry and harsh, and above natural temperature. I directed to be taken with the medicine, a drachm of wine of antimony every three hours, and a fourth of a grain of morphine at bed time, for the pain was continuous.

On the next afternoon, January 17th, I learned that the patient had rested quite well during the night, and since, had not felt any pain in the face; but was suffering as much as ever from a dull, heavy pain, pervading the head generally, and a sensation of roaring and buzzing in the ears. The medicine was omitted, and the patient took five grains of blue mass, and twelve of extract of colocynth. The cathartic acted three times before bed time, and the next day the patient was free from pain and unpleasant sensations in the head. There was no return of the disease, and the convalescence was rapid.

Case VI. Mrs. C., age 31. Nervous temperament, has been suffering from orbital neuralgia of the right side for six days, during which time she has taken freely of cathartics and anodynes.

I saw her the first time on the 10th of March, 1852, and found the patient suffering from the most excruciating pain. There was great intolerance of light and sound, twitching of the orbito-ocular groups of muscles; puffiness of the right side of the forehead and scalp. The eye of the affected side was almost closed from œdema. The pain was intense, lacerating and continuous. Bowels constipated. I directed an active purgative to be taken immediately, and after its action, to commence the use of the tincture of black snake root in teaspoonful doses every three hours.

I visited my patient the next day, and she expressed herself much relieved. Her bowels had acted freely, and she had taken half an ounce of the tincture. The medicine was continued; half an ounce more taken before the pain disappeared entirely; since when she has had no return of the disease. It may be of interest to add here, that Mrs. C. has been accustomed to neuralgic seizures during the cold and damp season of the year, and heretofore they have been exceedingly intractable.

CASE VII. Mrs. M., age 27, *enciente.* Sanguine temperament, has had facial neuralgia of right side, for seven days, during which time she has taken two ounces of the tincture of macrotys, prescribed by a druggist. The case is well marked, skin hot and dry, bowels constipated, pulse full with considerable force. I drew twelve ounces of blood from the arm, gave a saline cathartic, and ordered her to take a drachm of the tincture of macrotys, with as much wine of antimony every three hours.

The next day I learned the cathartic had acted freely, and the patient was free from pain. There was no return.

In presenting this summary, I have endeavored to select those cases which appeared to be purely nervous in their nature. I have also excluded those that were symptomatic of, and dependent upon local lesion going on in other parts; in order to exhibit the uniformity of action of this agent under certain circumstances. To secure the prompt therapeutic action of macrotys racemosa, it would appear important to relieve the alimentary canal of saburral secretions, and as far as possible, remove all irregularities of the circulating fluid. I do not know that its action tends in any manner to increase febrile reaction when present, nor to produce local determination to any particular organ; but on the contrary, I am strongly inclined to the belief, from my observation of the effects of this article, that it acts as a sedative to the heart's action. When excessive repletion or preternatural excitement of the heart and arteries is present, as in cases fourth and sixth, and others I might mention, the constitutional action of the remedy was not manifested until these conditions were removed. Neither did it act in subverting the disease. When I am now called upon to treat an idiopathic nervous affection, I prepare the system for the use of the cohosh, in the same manner as though I were going to administer quinine, to which I am strongly disposed to think it is related in its manner of action upon the nervous system. Where the system is thus prepared, in idiopathic attacks of neuralgia, I place more reliance in the above mentioned article, than any other of the materia medica. My confidence grows out of this fact: in my hands, it has acted more promptly in removing this painful affection, than any other remedy. It will not cure every case of *tic douleureux*, neither will quinine relieve every attack of fever and ague.

There are at least three features connected with the treatment of idiopathic neuralgia by black snake root, which will forcibly impress the mind of every one, who may chance to glance over the history of the above cases. I allude to the amenability of the disease to its action. The simplicity of the medication, and the speedy manner in which the use of the agent overcomes and subdues the painful affection. The only adjuvants that appear to be necessary to ensure its prompt action, are those calculated to remove general and local excitement of the vascular system, and correct the secretions. If the pain was severe, I might administer an *anodyne* to quiet the patient until the medicine had time to bring the system under its influence. It will be observed, that the time required to develop the constitutional effect of the remedy varies from twenty-four hours to three days, depending upon the state of the secretions, vascular system, and amount of medicine administered.

It is the nervous element of disease upon which the macrotys racemosa achieves its most salutary effects; and will be successful in controlling morbid action going on in that system, in proportion as it is uncomplicated. To this fact I am disposed to refer all the discrepancies of opinion which exist in the minds of authors, concerning the action of this remedial agent.

There was present in one half of the cases above mentioned, a certain train of cerebral symptoms, manifested by a feeling of uneasiness at first, speedily followed by a severe, dull, aching pain, pervading every part of the brain; together with a drawing at the external meatus, and a roaring, buzzing sound in the ears. I am unable to foretell what might have followed, had this medicine been pushed further in these cases, but I never have observed any symptoms of *narcotism*, mentioned by authorities as following the free administration of macrotys. I view this train of symptoms as so many indications of the constitutional effect or revolutionary action of the remedy. To this state of affairs, we may with propriety apply the term *macrotinism;* beyond which, it is neither necessary nor safe to go.

I am not prepared to say what effect macrotys may have upon the *nervous element* of intermittent fever, but reasoning *a priori*, I conclude that to test the *probability* is worthy of a patient and thorough trial.

